# Effect of the CB1 cannabinoid agonist WIN 55212-2 on the acquisition and reinstatement of MDMA-induced conditioned place preference in mice

**DOI:** 10.1186/1744-9081-6-19

**Published:** 2010-03-22

**Authors:** Carmen Manzanedo, Marta Rodríguez-Arias, Manuel Daza-Losada, Concepción Maldonado, María A Aguilar, José Miñarro

**Affiliations:** 1 Unidad de Investigación Psicobiología de las Drogodependencias, Departamento de Psicobiología, Facultad de Psicología, Universitat de Valencia, Avda. Blasco Ibáñez 21, 46010 Valencia, Spain

## Abstract

**Background:**

Numerous reports indicate that MDMA users consume other psychoactive drugs, among which cannabis is one of the most common. The aim of the present study was to evaluate, using the conditioned place preference, the effect of the cannabinoid agonist WIN 55,212-2 on the rewarding effects of MDMA in mice.

**Methods:**

In the first experiment adolescent mice were initially conditioned with 1.25, 2.5 or 5 mg/kg of MDMA or 0.1 or 0.5 mg/kg of WIN and subsequently with both drugs. Reinstatement of the extinguished preference by priming doses was performed in the groups that showed CPP. In the second experiment, animals were conditioned with 2.5 or 5 mg/kg of MDMA and, after extinction, reinstatement of the preference was induced by 0.5 or 0.1 mg/kg of WIN.

**Results:**

A low dose of WIN 55212-2 (0.1 mg/kg) increased the rewarding effects of low doses of MDMA (1.25 mg/kg), although a decrease in the preference induced by MDMA (5 and 2.5 mg/kg) was observed when the dose of WIN 55212-2 was raised (0.5 mg/kg). The CB1 antagonist SR 141716 also increased the rewarding effects of the lowest MDMA dose and did not block the effects of WIN. Animals treated with the highest WIN dose plus a non-neurotoxic dose of MDMA exhibited decreases of striatal DA and serotonin in the cortex. On the other hand, WIN 55212-2-induced CPP was reinstated by priming injections of MDMA, although WIN did not reinstate the MDMA-induced CPP.

**Conclusions:**

These results confirm that the cannabinoid system plays a role in the rewarding effects of MDMA and highlights the risks that sporadic drug use can pose in terms of relapse to dependence. Finally, the potential neuroprotective action of cannabinoids is not supported by our data; on the contrary, they are evidence of the potential neurotoxic effect of said drugs when administered with MDMA.

## Backgroud

Numerous reports indicate that MDMA users consume other psychoactive drugs, among which cannabis is one of the most common. Findings regarding concomitant abuse of MDMA and cannabis are similar in different countries, ranging from between 73% and 100% [for review see [1,2]]. Several studies of the prolonged combined use of MDMA and cannabis have highlighted an association with a variety of psychological problems, including elevated impulsiveness, anxiety and psychotic behavior [3].

MDMA is an indirect monoaminergic agonist, [4,5]. Cannabinoids exert their effect through interactions with specific endogenous CB1 and CB2 cannabinoid receptors [6,7] that are present in mammalian tissues. Many of the physiological responses provoked by MDMA are modulated by the endocannabinoid system [8,9]. This system interacts with a variety of neurotransmitters, including DA and 5-HT [9,10], and represents a common neurobiological substrate for the addictive properties of different drugs of abuse [11,12].

Both MDMA and cannabinoid agonists such as WIN 55212-2 (WIN) produce rewarding effects in mice [13,14] and rats when administered alone [15]. The few previous studies carried out to clarify the nature of the effects of exposure to cannabinoids on MDMA abuse liability, all of which were performed in rats, suggest that cannabinoid agonists potentiate the rewarding effects of MDMA [16] and that cannabinoid antagonists exhibit an opposite action [17]. However, a recent study by Robledo and co-workers [18] using mice has demonstrated that THC modifies the sensitivity of these animals to the behavioral effects of MDMA in different ways (increase/decrease) depending on the dose employed. The complex relation between cannabinoids and MDMA requires further study, especially regarding the potential mutual strengthening of their rewarding effects. The aim of the present study was to use the conditioned place preference (CPP) procedure to evaluate the influence of the cannabinoid agonist WIN 55212-2 on the rewarding effects of MDMA and the reinstatement of an extinguished preference in adolescent mice. This work may help to better understand the effects of polydrug abuse, particularly as we have chosen to study two of the most frequently used substances in adolescents. Firstly, we studied the rewarding effects of both drugs on the CPP and the ability of MDMA to reinstate the extinguished preference. The doses employed were chosen on the basis of previous work in our laboratory [13,14]. In order to assess whether or not the stimulation of the cannabinoid system increases the rewarding properties of MDMA, the effects of co-administration of WIN with rewarding and non-rewarding doses of MDMA were evaluated in the CPP model. The role of the CB1 cannabinoid receptor in the potentiating effects of WIN was tested using the CB1 antagonist SR 141716 (SR). In these experiments the ability of a priming dose (50% of that used for conditioning) of MDMA to reinstate the extinguished preference was evaluated. In a second set of experiments the ability of a priming dose of WIN to reinstate an MDMA-induced CPP (cross-reinstatement) was tested. Additionally, levels of brain monoamines and their metabolites were determined in the striatum, hippocampus and cortex of some of the experimental groups.

## Methods

### Subjects

Experiments were performed in a total of 265 male mice of the OF1 strain, of 21 days of age, acquired commercially from Charles River (Barcelona, Spain). They were housed in groups of four in plastic cages (25 × 25 × 14.5 cm) for 5 days before the experiments, under the following conditions: constant temperature, a reversed light schedule (white lights on: 19.30-07.30 h), and food and water available ad libitum, except during behavioral tests. Animals were handled over 2 days before the pre-conditioning (Pre-C) phase commenced to reduce their stress levels in response to experimental manipulations. The CPP began on PD 27 and the Post-C test was performed on PD38. Procedures involving mice and their care were conducted in compliance with national, regional and local laws and regulations, which are in accordance with the European Communities Council Directives (86/609/EEC, 24 November 1986).

### Apparatus

For place conditioning, we employed eight identical Plexiglas boxes with two equally sized compartments (30.7 cm length × 31.5 cm width × 34.5 cm height) separated by a gray central area (13.8 cm, length × 31.5 cm, width × 34.5 cm height). The compartments have different color walls (black vs white) and distinct floor textures (fine grid in the black compartment and wide grid in the white one). Four infrared light beams in each compartment of the box and six in the central area allowed the recording of the position of the animal and its crossings from one compartment to the other. The equipment was controlled by two IBM PC computers using MONPRE 2Z software (CIBERTEC, SA, Spain).

### Drugs and experimental design

Animals were injected i.p. with 1.25, 2.5 or 5 mg/kg of MDMA (± 3,4-methylenedioxymetamphetamine hydrochloride, Laboratorios Lipomed AG, Switzerland), 0.1 or 0.5 mg/kg of WIN (Tocris, Biogen Científica, S.L., Madrid, Spain), and 3 mg/kg of SR (Sanofi Recherche, Montpellier, France) in a volume of 0.01 ml/g. Control groups were injected with the physiological saline that was used to dissolve the drugs (NaCl 0.9%) or with Tween-80 (Sigma-Aldrich, Madrid, Spain), which was used to dissolve WIN and SR (two drops of Tween dissolve in saline).

### Procedure of CPP

#### Acquisition

Place conditioning consisted of three phases and took place during the dark cycle following an unbiased procedure in terms of initial spontaneous preference [for more details see [19]]. In brief, during pre-conditioning (Pre-C) mice were allowed access to both compartments of the apparatus for 900 s each day for 3 days. On day 3, the time spent by the animal in each compartment during a 900 s period was recorded. A total of 69 mice showed a strong unconditioned aversion (less than 33% of the session time) or preference (more than 67%) for one of the compartments and were, therefore, excluded from the study: 22 exp. 1; 9 exp.2; 24 exp. 3; 4 exp. 4; and 10 in exp. 5. In each group, half the animals received the drug or vehicle in one compartment and the other half in the other compartment. An ANOVA showed that there were no significant differences in the time spent in the drug-paired and the vehicle-paired compartments during the pre-conditioning phase. In the second phase (conditioning), animals were conditioned with saline, MDMA, WIN or both drugs through four pairings with the respective compartment (one pairing each day). Animals received an injection of the corresponding drug immediately prior to confinement in the drug-paired compartment for 1800 s on days 4, 6, 8 and 10, and received physiological saline before being confined to the vehicle-paired compartment for 1800 s on days 5, 7, 9 and 11. During the third phase, or post-conditioning (Post-C), which took place on day 12, the guillotine doors separating the two compartments were removed and the time spent by the untreated mice in each compartment during an observation period of 900 s was recorded.

#### Extinction

Conditioned groups underwent a daily extinction session which consisted of placing animals in the apparatus (without guillotine doors separating the compartments) for 900 s until the time spent in the drug-paired compartment by each group was similar to that of Pre-C and different from that of the Post-C test. Thus, all the animals in each group were submitted to the same number of extinction sessions, independently of their individual scores. Saline-conditioned groups underwent only one extinction session to confirm the lack of CPP. The extinction of CPP was always confirmed in a subsequent session 24 hours after the initial extinction session.

#### Reinstatement

The effects of a priming dose (in the first experiment half of that used for conditioning, and in the second experiment 0.1 or 0.5 mg/kg of WIN) were evaluated 24 hours after confirmation of extinction. The reinstatement test was the same as that for Post-C (free ambulation for 900 s), except that animals were tested 900 s after administration of the respective dose of the drug.

### Experiment 1: CPP induced by MDMA plus the CB1 cannabinoid agonist WIN 55212-2

To evaluate if the rewarding effects of MDMA could be increased by the CB1 cannabinoid agonist WIN, animals were conditioned during the conditioning phase with: saline (SAL), 1.25, 2.5 or 5 mg/kg of MDMA (MDMA 1.25; MDMA 2.5; MDMA 5), 0.1 or 0.5 mg/kg of WIN 55212-2 (WIN 0.1, WIN 0.5), 1.25, 2.5 or 5 mg/kg of MDMA plus 0.1 mg/kg of WIN (MDMA 1.25 + WIN 0.1; MDMA 2.5+ WIN 0.1; MDMA 5+ WIN 0.1) or 1.25, 2.5 or 5 mg/kg of MDMA plus 0.5 mg/kg of WIN (MDMA 1.25 + WIN 0.5; MDMA 2.5+ WIN 0.5; MDMA 5+ WIN 0.5). In the groups showing preference, reinstatement was induced after extinction with a priming dose of MDMA (half of that used for conditioning). In a new set of animals conditioned with saline, reinstatement was induced with 0.6, 1.25 and 2.5 mg/kg of MDMA.

Four new groups were employed in order to evaluate the putative role of the CB1 cannabinoid receptor in the potentiating effects of WIN on the MDMA-induced CPP. During the conditioning phase, animals received 5 mg/kg of MDMA plus 3 mg/kg of SR alone or plus 0.1 mg/kg of WIN (MDMA 5 + SR 3; MDMA 5 + SR 3 + WIN 0.1); or 1.25 mg/kg of MDMA plus 3 mg/kg of SR alone or plus 0.1 mg/kg of WIN (MDMA 1.25 + SR 3; MDMA 1.25 + SR 3 + WIN 0.1).

### Experiment 2: Cross reinstatement by WIN 55212-2 of MDMA-induced CPP

To evaluate if the MDMA-induced CPP was reinstated by WIN, animals were conditioned during the conditioning phase with 2.5 or 5 mg/kg of MDMA and, after extinction, reinstatement of the preference was induced by 0.5 or 0.1 mg/kg of WIN (MDMA 2.5-RWIN 0.5; MDMA 5-RWIN 0.5; MDMA 2.5-RWIN 0.1; MDMA 5-RWIN 0.1; Sal-RWIN 0.1; Sal-RWIN 0.1).

### Analysis of biogenic amines

Nine separate groups of animals underwent the same schedule of treatment as applied in the conditioning sessions, i.e. four injections on alternate days (intermittent schedule) of saline (Sal), 1.25 or 5 mg/kg of MDMA (M1.25; M5); 0,1 or 0,5 mg/kg of WIN (W 0.1; W 0.5); and 1.25 or 5 mg/kg of MDMA plus 0,1 or 0,5 mg/kg of WIN (M 1.25 + W 0.1; M 5 + W 0.5; M 1.25 + W 0.1 M 1.25 + W 0.1. At the time of the test, 48 hours (intermittent schedule) after the last injection, mice were sacrificed by cervical fracture. Monoamines were analyzed in a high performance liquid chromatograph (Agilent 1100 series HPLC) following the methodological procedure described in Daza-Losada et al. [14]. Dopamine (DA), dihydroxyphenyl acetic acid (DOPAC) and homovanilic acid (HVA) were analyzed in the striatum and serotonin (5-HT), and 5-hidroxyindole acetic acid (5-HIAA) was analyzed in the striatum, cortex and hippocampus.

### Statistical analysis

To evaluate the acquisition of CPP, the data of the time spent by the animals in the drug-paired compartment were analyzed with a mixed ANOVA with two between subject variables - Dose of WIN, with three levels (0, 0.1 and 0.5), and Dose of MDMA, with three levels (1.25, 2.5 and 5) - and one within variable - Days, with two levels (Pre-C and Post-C). Bonferroni adjustment was employed for post hoc comparisons.

In the case of the groups which developed preference, each of the MDMA doses employed was submitted to a new MANOVA with one between subject variable - "treatment", with two levels for the doses of MDMA 1.25 and 2.5 mg/kg (M1.25+WIN0.01 and M1.25+ SR+ WIN0.1; M2.5 and M2.5 + WIN0.1) and four levels for the dose of 5 mg/kg (M5, M5+WIN0.1, M5+ SR, and M5+ SR + WIN0.1) - and one within-subject variable - "Days", with four levels (Pre-C, Post-C, extinction and reinstatement). In all the analyses, the Bonferroni adjustment was employed to make post hoc comparisons. In the group conditioned with 0.5 mg/kg of WIN, differences between the time spent in the drug-paired compartment in Pre-C and Post-C and in each extinction session or reinstatement test were analyzed by means of a Student's t-test.

Similar analyses were performed in order to evaluate the role of the CB1 receptors in the effects of WIN on the acquisition and reinstatement of the MDMA-induced CPP. The data of the time spent in the drug-paired compartment were analyzed with a mixed ANOVA with two between subject variables - Dose of MDMA, with two levels (1.25 and 5 mg/kg), and Cannabinoid treatment, with three levels (Saline, SR and WIN 0.1) - and one within variable - Days, with two levels (Pre-C and Post-C). In the case of the groups which developed preference, a new MANOVA was performed with one within-subject variable: "Days", with four levels (Pre-C, Post-C, extinction and reinstatement). Bonferroni adjustment was employed for post hoc comparisons.

To evaluate if the MDMA-induced CPP was reinstated by WIN, the data of the time spent in the drug-paired compartment were analyzed with a mixed ANOVA with two between subject variables - Dose of MDMA, with three levels (0, 2.5 and 5 mg/kg), and Dose of WIN, with three levels (0, 0.1 and 0.5 mg/kg) - and one within variable - Days, with four levels (Pre-C, Post-C, extinction and reinstatement). Bonferroni adjustment was employed for post hoc comparisons.

Biogenic amines were analyzed with a mixed ANOVA with two between subject variables: MDMA dose, with three levels (0, 1,25 and 5 mg/kg), and WIN dose, with three levels (0, 0,1 and 0,5 mg/kg). In all the analyses, the Bonferroni adjustment was employed for post hoc comparisons.

## Results

### Experiment 1: CPP induced by MDMA and the CB1 cannabinoid agonist WIN 55212-2

**Figure 1:**
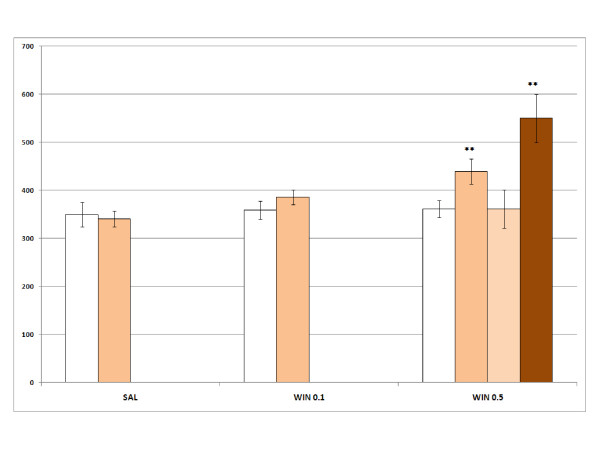
**Acquisition, extinction and reinstatement of WIN-induced CPP**. Acquisition, extinction and reinstatement of WIN-induced CPP in three groups of animals: SAL, animals receiving saline in both compartments (n = 10); WIN 0.1, WIN 0.5, animals receiving 0.1 or 0.5 mg/kg of WIN in the drug-paired compartment (n = 10). The bars represent the mean (± SEM) time spent in the drug-paired compartment before conditioning sessions (white bars), after conditioning sessions (orange bars), in the last extinction session (light orange bars) and in the reinstatement test (brown). ** p < 0.02 significant difference in the time spent in pre-conditioning vs. post-conditioning sessions or reinstatement tests.

**Figure 2:**
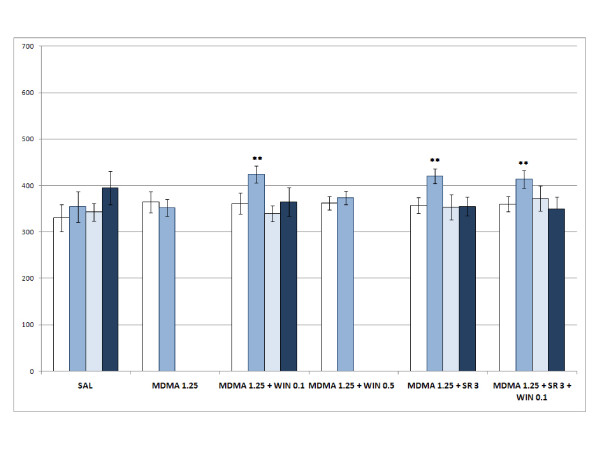
**Acquisition, extinction and reinstatement of 1.25 mg/kg MDMA- and WIN-induced CPP**. Acquisition, extinction and reinstatement of MDMA- and WIN-induced CPP in six groups of animals: SAL, animals receiving saline in both compartments (n = 10); MDMA 1.25, animals receiving 1.25 mg/kg of MDMA in the drug-paired compartment (n = 10); MDMA 1.25 + WIN 0.5 and MDMA 1.25 + WIN 0.1 animals receiving 0.5 or 0.1 mg/kg of WIN plus 1.25 mg/kg of MDMA in the drug-paired compartment (n = 11); MDMA 1.25 + SR 3 + WIN 0.1, animals conditioned with 1.25 mg/kg of MDMA plus 3 mg/kg of SR plus 0.1 mg/kg of WIN (n = 14). The bars represent the mean (± SEM) time spent in the drug-paired compartment before conditioning sessions (white bars), after conditioning sessions (blue bars), in the last extinction session (light blue bars) and in the reinstatement test (dark blue bars). ** p < 0.02 significant difference in the time spent in pre-conditioning vs. post-conditioning sessions or reinstatement tests.

**Figure 3:**
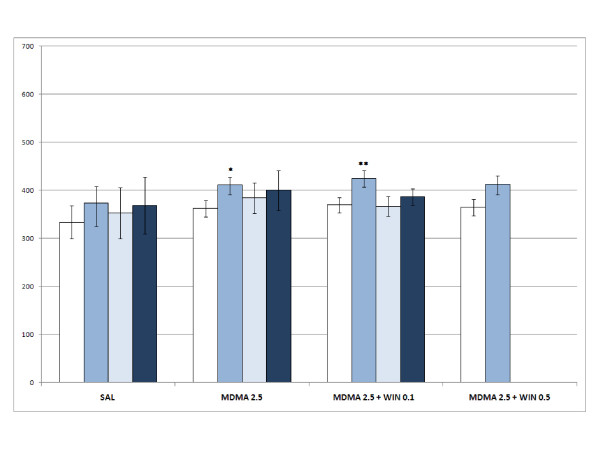
**Acquisition, extinction and reinstatement of 2.5 mg/kg MDMA- and WIN-induced CPP**. Acquisition, extinction and reinstatement of MDMA- and WIN-induced CPP in four groups of animals: SAL, animals receiving saline in both compartments (n = 10); MDMA 2.5, animals receiving 2.5 mg/kg of MDMA in the drug-paired compartment (n = 10); MDMA 2.5 + WIN 0.5 and MDMA 2.5 + WIN 0.1 animals receiving 0.5 or 0.1 mg/kg of WIN plus 2.5 mg/kg of MDMA in the drug-paired compartment (n = 11). The bars represent the mean (± SEM) time spent in the drug-paired compartment before conditioning sessions (white bars), after conditioning sessions (blue bars), in the last extinction session (light blue bars) and in the reinstatement test (dark blue bars).  * p < 0.05; ** p < 0.02 significant difference in the time spent in pre-conditioning vs. post-conditioning sessions or reinstatement tests.

**Figure 4:**
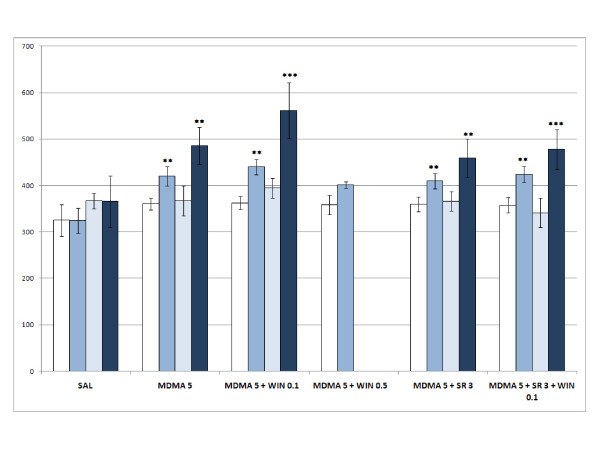
**Acquisition, extinction and reinstatement of 5 mg/kg MDMA- and WIN-induced CPP**. Acquisition, extinction and reinstatement of MDMA- and WIN-induced CPP in six groups of animals: SAL, animals receiving saline in both compartments (n = 10); MDMA 5, animals receiving 5 mg/kg of MDMA in the drug-paired compartment (n = 10); MDMA 5 + WIN 0.5 and MDMA 5 + WIN 0.1 animals receiving 0.5 or 0.1 mg/kg of WIN plus 5 mg/kg of MDMA in the drug-paired compartment (n = 11); MDMA 5 + SR 3 + WIN 0.1, animals conditioned with 5 mg/kg of MDMA plus 3 mg/kg of SR plus 0.1 mg/kg of WIN (n = 14). The bars represent the mean (± SEM) time spent in the drug-paired compartment before conditioning sessions (white bars), after conditioning sessions (blue bars), in the last extinction session (light blue bars) and in the reinstatement test (dark blue bars). ** p < 0.02; *** p < 0.002 significant difference in the time spent in pre-conditioning vs. post-conditioning sessions or reinstatement tests.

The ANOVA for acquisition revealed a significant effect of the variable Days [F(1,119) = 18.043; p < 0.001], as more time was spent in the drug-paired compartment during the Post-C test (p < 0.001), and the Interaction Days × WIN × Dose of MDMA [F(6,119) = 2.454; p < 0.028]. Only the groups WIN 0.5 (Fig. 1), MDMA 1.25+WIN 0.1 (Fig. 2), MDMA 2.5 (Fig. 3), MDMA2.5+WIN0.1 (Fig. 3), MDMA 5 (Fig. 4), and MDMA 5+ WIN 0.1 (Fig. 4) developed CPP, as they spent more time in the drug-paired compartment during the Post-C test than in the Pre-C test (p < 0.05 for MDMA 2.5 and p < 0.02 for the rest of the groups). In the Post-C test, significantly higher scores were obtained by the MDMA 5 group with respect to the SAL group and by the WIN 0.5 group with respect to the MDMA 1.25 + WIN 0.5 group (p < 0.05 and p < 0.02, respectively). Thus, CPP was obtained with MDMA doses equal to or higher than 2.5 mg/kg and with any MDMA dose plus 0.1 mg/kg of WIN. The highest dose of WIN (0.5 mg/kg) also induced CPP.

Among the groups showing preference, the number of sessions required for extinction was 13 in the MDMA 5 + SR 3 + WIN 0.1 group, 11 in the MDMA 5 + WIN 0.1 group, 6 in the MDMA 2.5 + WIN 0.1 group, 3 in the MDMA 2.5 and MDMA 5 + SR 3 groups, 4 in the MDMA 5 group, 2 in the WIN 0.5 and MDMA 1.25 + WIN 0.1 groups, and 1 in the MDMA 1.25 + SR 3 + WIN 0.1 group.

The MANOVA performed for the groups in which reinstatement was induced by 0.625 and 1.25 mg/kg of MDMA revealed that reinstatement of this preference was not achieved in any of the groups (Fig. 2 and 3).

The MANOVA performed for the groups in which reinstatement was induced by 2.5 mg/kg of MDMA (Fig. 4) revealed a significant effect of the variable Days [F(3,52) = 14.494; p < 0.001] and the interaction Days × Treatment [F(12,162) = 1.866; p < 0.04], with reinstatement of the preference observed in all the groups (p < 0.02 for MDMA5 and MDMA 5+ SR 3 + WIN 0.1, and p < 0.002 for the rest of the groups) with the exception of that conditioned with saline and treated with 2.5 mg/kg of MDMA as a priming dose. Thus, only in mice in which CPP was induced with 5 mg/kg of MDMA was reinstatement of the preference observed.

The AVOVA for the effects of SR revealed a significant effect of the variables Days [F(1,68) = 23.206; p < 0.001], the interaction Days × Dose of MDMA [F(1,68) = 5.621; p < 0.021], and the interaction Days × Dose of MDMA × Cannabinoid treatment [F(2,68) = 4.536; p < 0.014]. All the groups, except MDMA 1.25, developed CPP (p < 0.02 for MDMA 1.25 +SR 3 (Fig. 2), MDMA 1.25 +SR 3 +WIN 0.1 (Fig. 2), and MDMA 5 +SR 3 (Fig. 4); and p < 0.001 for the rest). Animals treated with MDMA 1.25 +SR 3 or MDMA 1.25 +SR 3 +WIN 0.1 (Fig. 2) presented higher scores in the Post-C test than those treated only with MDMA 1.25 (p < 0.003). Additionally, the MDMA 5 group obtained a higher score in the Post-C test than the MDMA 1.25 group (p < 0.001), with no differences being observed between the other groups. In general, SR increased the reinforcing effects of MDMA and did not block the effects of WIN.

The MANOVA for the effect of SR on the reinstatement of a MDMA-induced CPP revealed an effect of the variable Days [F(3,55) = 17.814; p < 0.001] and the interaction Days × Groups of treatment [F(4.57) = 4.697; p < 0.02], as the extinguished preference was reinstated only in the groups conditioned wit 5 mg/kg of MDMA (Fig. 4), regardless of the other treatment received (p < 0.02 for MDMA 5 +SR 3, and p < 0.001 for MDMA 5 +SR 3 + WIN 0.1).

### Experiment 2: Cross reinstatement of MDMA-induced CPP by WIN 55212-2

**Figure 5:**
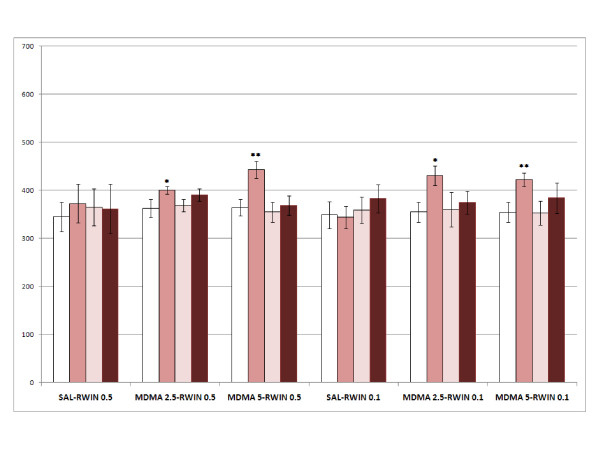
**Reinstatement of MDMA-induced CPP by priming injections of WIN**. Reinstatement of MDMA-induced CPP by priming injections of WIN in six groups of animals (n = 10): Sal-RWIN 0.5 and Sal-RWIN 0.1, animals conditioned with saline in the drug-paired compartment and receiving a priming injection of 0.5 or 0.1 mg/kg of WIN; MDMA 2.5-RWIN 0.5 and MDMA 2.5-RWIN 0.1, animals conditioned with 2.5 mg/kg of MDMA in the drug-paired compartment and receiving a priming injection of 0.5 or 0.1 mg/kg of WIN.; MDMA 5-RWIN 0.5 and MDMA 5-RWIN 0.1, animals conditioned with 5 mg/kg of MDMA in the drug-paired compartment and receiving a priming injection of 0.5 and 0.1 mg/kg of WIN. The bars represent the mean (± SEM) time spent in the drug-paired compartment before conditioning sessions (white bars), after conditioning sessions (pink bars), in the last extinction session (light pink bars) and in the reinstatement test (dark pink bars). * = p < 0.05, ** = p < 0.02 significant difference in the time spent in pre-conditioning vs. post-conditioning sessions or reinstatement tests.

The results obtained in Experiment 2 are represented in Fig. 5.

The ANOVA revealed a significant effect of the variable Days [F(3,56) = 9.667; p < 0.001] and the Interaction Days × Dose of MDMA [F(3,56) = 217; p < 0.019], Post-C test scores significantly higher than that of the Pre-C test in the groups conditioned with 2.5 or 5 mg/kg of MDMA (p < 0.05 and p < 0.02, respectively). Reinstatement of this preference was not achieved in any of the groups.

#### Brain monoamines

The ANOVA for the striatal levels of DA revealed a significant effect of the variable MDMA dose [F(4, 49) = 3.530; p < 0.037], as lower levels of DA were detected in the groups treated with 5 mg/kg of MDMA than in those treated with 1.25 mg/kg of MDMA (p < 0.05). The interaction WIN dose × MDMA dose [F(4, 49) = 2.791; p < 0.036] also had a significant effect, producing lower concentrations of DA in the M5+WIN 0.5 group than in the Sal and M1.25 +Win 0.5 groups (p < 0.05 and p < 0.001, respectively). No differences were observed in the concentrations of DOPAC, HVA, serotonin or 5-HIIA in the striatum (see Additional file 1).

In the cortex, serotonin levels showed a significant effect for the variable MDMA dose [F(2,46) = 9.141; p < 0.001] and the interaction MDMA dose × WIN dose [F(4,46) = 4.798; p < 0.003]. Groups treated with 5 or 1.25 mg/kg of MDMA plus 0.5 mg/kg of WIN exhibited lower levels of serotonin than those treated only with this dose of WIN (W0.5). In addition, the M1.25 + W 0.5 group displayed lower serotonin levels than the M1.25 + W 0.1 (p < 0.002) group. The levels of 5-HIAA in the cortex showed a significant effect of the variable MDMA dose [F(2,45) = 5.285; p < 0.008], which was lower in the groups treated with 5 mg/kg of MDMA (p < 0.05), and for the variable WIN dose [F(2,45) = 3.394; p < 0.04], which was also lower in the groups receiving the highest WIN dose (0.5 mg/kg).

In the hippocampus, serotonin concentration had significant effects on the interaction of the variables MDMA dose × WIN dose [F(4,46) = 2.828; p < 0.03]. Animals treated with 5 mg/kg of MDMA alone (M5) exhibited higher levels than those treated with saline (Sal) (p < 0.05). 5-HIAA levels had significant effects on the interaction of the variables MDMA dose × WIN dose [F(4,44) = 3.271; p < 0.01]. In the groups treated with MDMA (M5 and M1.25), the concentration of this metabolite was lower than in the group treated with saline (p < 0.05). The groups treated with the lowest dose of MDMA plus any of the WIN doses (M1.25 + W0.1 or M1.25 + W0.5) displayed higher levels of 5-HIAA than those treated only with 1.25 mg/kg of MDMA (M1.25) (p < 0.01).

## Discussion

The results of the present study confirm the complex interaction between the cannabinoid system and MDMA. The cannabinoid agonist WIN 55212-2 can increase or decrease the acquisition of MDMA-induced CPP depending on the dose employed. The CB1 receptor is not involved in the potentiating effect of WIN. We have also observed cross-reinstatement, since MDMA was capable of reinstating the WIN-induced CPP. Additionally, in contrary to the presumed neuroprotective role of cannabinoids, the present results show that WIN 55212-2 induces a decrease in the concentrations of dopamine in the striatum and serotonin in the cortex when administered with a non-neurotoxic dose of MDMA.

We have confirmed that MDMA is capable of inducing CPP even at low doses (2.5 mg/kg), and that reinstatement of the CPP can be achieved with priming doses 50% lower than that used for conditioning. In addition, the CB1 agonist WIN also induced CPP with the highest dose employed (0.5 mg/kg). Cross-reinstatement was observed, since the WIN-induced preference was reinstated after a priming dose of MDMA (5 mg/kg). The results obtained in these experiments confirm and extend those of a previous report by our group [14]. On the other hand, the induction of place preference with cannabinoid agonists and antagonists has been reported in several studies [20-23]. Specifically, WIN 55212-2 induces CPP when administered peripherically [13] or injected into the hippocampus [15]. We have no knowledge of previous reports of cross-reinstatement between a cannabinoid agonist-induced CPP and MDMA. The strength of this reinstatement in the WIN-induced CPP group was similar to that in the group conditioned with MDMA, or even higher. Moreover, more time was required for the reinstated preference to be extinguished in mice conditioned with the cannabinoid agonist. The most important implication of the cross reinstatement observed is that exposure to MDMA can produce an intense craving and relapse to drug-taking behavior in cannabinoid-dependent humans.

On the other hand, the MDMA-induced CPP was not reinstated by priming injections of WIN (second experiment). Thus, cross reinstatement was not observed in this case. One possible justification for this lack of reciprocity is that these drugs have a particular neurotransmitter activation profile. MDMA is a DA releaser that affects other systems, such as the serotoninergic system, but cannabinoids seem to play a modulating role in DA transmission. Another possible explanation is that, in animals conditioned with MDMA, the first exposure to the cannabinoid agonist induces an aversive effect that does not recall the preference induced by MDMA. Conditioned place aversion induced by WIN has previously been reported [24]. We have recently induced reinstatement of MDMA-induced CPP by priming doses of WIN (0.1 mg/kg) in mice previously exposed to this cannabinoid agonist [25], which gives support to the latter hypothesis.

On the other hand, the CB1 agonist WIN exerted a dual effect on the MDMA-induced CPP. The highest WIN dose (0.5 mg/kg), which in itself induced CPP, diminished preference when administered with an effective dose of MDMA (2.5 and 5 mg/kg). On the other hand, a low dose of this CB1 agonist (0.1 mg/kg), inactive when administered alone, strengthened the rewarding effects of an inactive dose of MDMA (1,25 mg/kg) and induced CPP. Moreover, the time needed for the preference to be extinguished was longer in the groups treated with the lowest dose of WIN 55212-2. Our results are in accordance with those of Robledo et al [18], who reported that a sub-threshold dose of THC produced CPP in mice when combined with a non-rewarding dose of MDMA (3 mg/kg), but decreased the CPP induced by an effective dose of MDMA (10 mg/kg).

Self-administration studies have also confirmed that the endogenous cannabinoid system influences the mechanism that regulates MDMA's rewarding effect. Braida and Sala [16] demonstrated that the cannabinoid agonist CP 55,940 altered i.c.v. MDMA self-administration and significantly reduced MDMA intake, suggesting a synergistic action of cannabinoid agonists on the rewarding properties of MDMA. On the other hand, in a report by Robledo et al [18], the heightened operant response induced by the cannabinoid antagonist SR in MDMA self-administration indicated a decrease in sensitivity to the motivation for obtaining the drug. In addition, THC increased the threshold for MDMA when self-administrated.

Cannabinoids participate in the regulation of dopamine synthesis, release and turnover [26]. The overlapping expression of cannabinoid and dopamine receptors in some brain areas, such as the NAC [27], may represent a neuroanatomical substrate for such an interaction. The complex relation between cannabinoids and MDMA is also observed in microdialysis studies of DA release into the NAC. A low dose of THC was shown to significantly increase DA outflow in the NAC, while a low dose of MDMA did not. When MDMA was administered before THC, DA levels were lower with respect to those of THC. However, when THC was administered before MDMA, DA levels were not significantly modified with respect to those of THC [18]. If we apply these results to our experiment in which both drugs (MDMA and WIN) were administered together, we can hypothesize that doses of MDMA or WIN that in themselves can increase DA release would exert an antagonistic action on DA; i.e, DA would not be released.

The cannabinoid-selective CB1 antagonist SR 141716 did not affect the CPP or reinstatement of the preference induced by 5 mg/kg of MDMA when administered alone or with WIN. In the groups conditioned with 1.25 mg/kg of MDMA, which was not effective by itself, CPP was observed when administered with the CB1 antagonist. Moreover, when this sub-threshold dose of MDMA was administered with 0.1 mg/kg of WIN, the CPP induced was not affected by blockade of the CB1 receptor. These results can be explained if the sense that the CB1 cannabinoid agonist WIN exerts its effects in a way that does not involve the CB1 receptors. Although WIN is a CB1 receptor agonist, some of its effects seem to be mediated by mechanisms that do not involve cannabinoid receptors, and which could be calcium-dependent mechanisms [28]. On the hand, SR141716 is capable of producing conditioned place preference in rats [29,21], although studies performed in our laboratory failed to detect an motivational action of this CB1 antagonist [13]. In a similar way to WIN, SR 141716 is reported to exhibit an independent CB1 activity; for instance, it exerts an anxiolytic effect on CB1 knockout mice [30] The aversive effects of cannabinoid agonists observed in many studies, and the rewarding effect of cannabinoid antagonists are suggestive of a cannabinergic tone in the rat brain. Cannabinoid receptors, while present in areas of the brain that support electrical self-stimulation, such as the nucleus accumbens or the lateral septum [31], are also localized in the central and basolateral amygdaloid nuclei and in the periaqueductal gray, two areas that support electrically-induced fear responses and are thought to be the sites of action of certain anxiogenic drugs. The observation by Herkenham and Brady [32] that D9-THC induces c-Fos expression in stress-responsive nuclei of the rat brain further supports an anxiogenic role of cannabinoids. Recently, Wu and co-workers [33] have demonstrated that SR141716A employed at the dose used in the present study (3 mg/kg, i.p.) produces an increase of DA release in the dorsal and ventral striatum, although inhibition of dopamine release induced by nicotine, ethanol, and cocaine has been described for other authors [34]. The present data suggest that endogenous cannabinoids both inhibit reward and induce aversion by actions exerted within these circuits [29].

The role of CB1 cannabinoid receptors in the rewarding effects of drugs of abuse is not altogether clear [24,35-37]. Although SR reduces the acquisition of CPP induced by cocaine, morphine or food in a dose-depend manner, it does not affect the expression of the cocaine-induced CPP [24]. More recently, studies performed in CB1 knockout mice [38] have shown that they develop a normal CPP despite the fact that many of the effects of MDMA are diminished in these animals, among them self-administration. The CB1 cannabinoid receptor is essential in the acute rewarding effects of several drugs of abuse that produce their effect on the ventral tegmental area, such as nicotine, ethanol and morphine. However, psychostimulant drugs produce their effect directly on the nucleus accumbens DA terminals [11]. For that reason, the CB1 cannabinoid receptor does not seem to contribute to their acute rewarding effects when evaluated in the CPP. Accordingly, previous studies have reported that cocaine-induced CPP but not self-administration is preserved in CB1 knockout mice [39]. In the study of Braida et al [17], SR blocked the MDMA-induced CPP, although there are several and important differences between this and the present study, including the species used and the form of MDMA administration (intracerebroventricular). Moreover, SR increased the rewarding effects of 1.25 mg/kg MDMA. We can hypothesize that, when SR is administered jointly with WIN, the antagonist blocks the CB1 receptor and in that way inhibits WIN action, while at the same time decreasing the cannabinoid tone and indirectly increasing the rewarding action of MDMA.

As we have previously observed [14], MDMA did not affect the concentration of DA or serotonin, or their metabolites in the striatum or cortex at the doses employed during the CPP procedure. However, an increase in serotonin concentration (5 mg/kg) and decreases in the levels of 5-HIAA were observed in the hippocampus. The CB1 agonist WIN did not affect the brain monoamines, with the exception of an increase in the level of 5-HIAA in the hippocampus with the lowest dose (0.1 mg/kg). Nevertheless, in the striatum of the animals treated with the highest doses of MDMA and WIN, a decrease in the DA concentration (over 40%) was observed. A reduction in cortical serotonin occurred in the same group (over 28%) and in the groups treated with the highest dose of MDMA plus WIN 0.1 (in which there was also an increase in the levels of 5-HIAA in the hippocampus) or the lowest dose of MDMA plus the highest dose of WIN (over 20% of a reduction of serotonin in both groups).

The anti-inflamatory and anti-oxidative properties of cannabinoids point to their possible beneficial effects with respect to limiting neurological damage, particularly by decreasing neuroinflamation [40,41]. However, animal studies have revealed that chronic administration of the cannabinoid THC causes hippocampal damage [42], and several reports have highlighted that exposure to low concentrations of cannabinoids over a prolonged period is likely to have a neurotoxic effect [43,44]. Acute administration at high concentrations of cannabinoids at the time of trauma, on the other hand, may be neuroprotective. The decrease in striatal DA and cortical serotonin in animals treated with 5 mg/kg of MDMA plus the highest dose of WIN (0.5 mg/kg) could explain the surprising lack of CPP observed in this group. With a daily conditioning schedule we have seen that 5 mg/kg of MDMA induces a similar decrease in cortical serotonin and striatal DA and does not achieve preference in the Post-C test [14]. However, these results should be interpreted with caution, as the measurements were obtained only 48 h after the last drug administration.

## Conclusions

The results of the present study confirm that the cannabinoid system plays a role in the rewarding effects of MDMA. A high proportion of MDMA users also consume cannabis, which makes the study of the interaction between the two drugs highly relevant. Moreover, the observed cross-reinstatement between these drugs highlights the importance of sporadic drug use in relapse to dependence. To conclude, our results do not support a neuroprotective action of cannabinoids; the present data show that WIN 55212-2 induces a decrease in the concentrations of dopamine and serotonin in the striatum and cortex 48 hours after administration in conjunction with a non-neurotoxic dose of MDMA.

## Competing interests

The authors declare that they have no competing interests.

## Authors' contributions

CM carried out part of the CPP studies. MR-A designed the experimental procedures, performed the statistical analyses and drafted the manuscript. MD-L carried out part of the CPP studies. CMaldonado performed the HPLC analyses. MAA designed of the experimental procedures and drafted the manuscript. JM participated in the design of the study and helped to draft the manuscript. All authors have read and approved the final manuscript.

## Supplementary Material

Additional file 1 Effects of MDMA or WIN administration on the concentrations of monoamines (ng/mg tissue) in the striatum, cortex and hippocampus. Click here for file
