# Supplementary material for: Effect of the CB1 cannabinoid agonist WIN 55212-2 on the acquisition and reinstatement of MDMA-induced conditioned place preference in mice
Source: Behav Brain Funct. 2010 Mar 22;6:19. doi: 10.1186/1744-9081-6-19 (PMC2858089; doi:10.1186/1744-9081-6-19)
Supplement: Additional file 1 — Effects of MDMA or WIN administration on the concentrations of monoamines (ng/mg tissue) in the striatum, cortex and hippocampus. [file 1744-9081-6-19-S1.DOC]

**Table 1. Effects of MDMA or WIN administration on the concentrations of monoamines (ng/mg tissue) in the striatum, cortex and hippocampus**

|  | **Sal** | **M5** | **M1.25** | **W0.5** | **W0.1** | **M5 + W0.5** | **M5 + W0.1** | **M1.25 + W0.5** | **M1.25 + W0.1** |
| --- | --- | --- | --- | --- | --- | --- | --- | --- | --- |
| *Striatum* |  |  |  |  |  |  |  |  |  |
| **DA** | 12237 ± 1673 | 9835 ± 1018 | 10212 ± 861 | 10678 ± 1402 | 11041 ± 1046 | 7549 ± 699 * | 10727 ± 910 | 15203 ± 910 | 11453 ± 1038 |
| **DOPAC** | 1134 ± 197 | 1928 ± 564 | 1329 ± 362 | 1487 ± 268 | 1501 ± 195 | 4173 ± 417 | 1594 ± 314 | 2997 ± 493 | 2297 ± 483 |
| **HVA** | 140 ± 14 | 196 ± 74 | 162 ± 15 | 119 ± 12 | 125 ± 15 | 150 ± 9 | 157 ± 12 | 167 ± 22 | 126 ± 5 |
| **5-HT** | 761 ± 130 | 759 ± 112 | 789 ± 128 | 653 ± 176 | 537 ± 44 | 527 ± 23 | 560 ± 82 | 486 ± 54 | 517 ± 44 |
| **5-HIAA** | 490 ± 67 | 559 ± 131 | 448 ± 102 | 533 ± 53 | 432 ± 52 | 580 ± 35 | 427 ± 37 | 489 ± 65 | 442 ± 79 |
|  |  |  |  |  |  |  |  |  |  |
| *Cortex* |  |  |  |  |  |  |  |  |  |
| **5-HT** | 274 ± 13 | 258 ± 7 | 259 ± 11 | 298 ± 5 | 275 ± 21 | 200 ± 3 | 219 ± 82 | 219 ± 12 | 299 ± 15 |
| **5-HIAA** | 363 ± 5 | 307 ± 10 | 330 ± 14 | 327 ± 5 | 288 ± 28 | 227 ± 7 | 302 ± 21 | 326 ± 22 | 321 ± 25 |
|  |  |  |  |  |  |  |  |  |  |
| *Hippocampus* |  |  |  |  |  |  |  |  |  |
| **5-HT** | 1219 ± 324 | 1904 ± 223 * | 1830 ± 107 | 1154 ± 299 | 1855 ± 158 | 1445 ± 217 | 1335 ± 300 | 1666 ± 106 | 2128 ± 196 |
| **5-HIAA** | 473 ± 81 | 222 ± 63 * | 102 ± 37 * | 372 ± 89 | 286 ± 39 | 273 ± 19 | 397 ± 54 | 366 ± 60 | 318 ± 12 |

Effects of MDMA or WIN administration on the concentrations of monoamines (ng/mg tissue) in the striatum, cortex and hippocampus following an alternating administration schedule in nine groups of animals: Sal, animals receiving saline (n=10); M5, M1.25 animals receiving 5 or 1.25 mg/kg of MDMA (n=); W0.1, W0.5, animals receiving 0.1 or 0.5 mg/kg of WIN (n=10); M5 + W0.1, M5 + W0.5, animals receiving 5 mg/kg of MDMA plus 0.1 or 0.5 mg/kg of WIN (n=10); M1.25 + W0.1, M1.25 + W0.5, animals receiving 1.25 mg/kg of MDMA plus 0.1 or 0.5 mg/kg of WIN (n=10). Animals received 4 injections of the

corresponding drug (or drugs) every 48 hours for 8 days. Amine levels were evaluated 48 hours after the last injection. * p<0.05 significant difference with respect to saline grou
